# *Notes from the Field:* Syndromic Surveillance Used To Monitor Emergency Department Visits During a Synthetic Cannabinoid Overdose Outbreak — Connecticut, August 2018

**DOI:** 10.15585/mmwr.mm6908a4

**Published:** 2020-02-28

**Authors:** Sydney A. Jones, Kristen Soto, Erin Grogan, Alexander Senetcky, Susan Logan, Matthew Cartter

**Affiliations:** ^1^Epidemic Intelligence Service, Division of Scientific Education and Professional Development, Center for Surveillance, Epidemiology, and Laboratory Services, CDC; ^2^Connecticut Department of Public Health.

On the morning of August 15, 2018, the Connecticut Department of Public Health (CTDPH) learned from media reports about multiple persons found unresponsive in a city park in New Haven County after using synthetic cannabinoids (SCs), manmade psychoactive substances that can cause unpredictable and sometimes severe health effects. Prevalence of acute SC poisonings has increased in the United States in recent years (*1*). Syndromic surveillance data collected in near real-time have been used to track outbreaks of illness and to improve public health authorities’ situational awareness about trends in suspected drug overdoses (*2*). CTDPH monitored syndromic surveillance data from emergency department (ED) visit records to identify the magnitude of the SC overdose outbreak and provide situational awareness during the outbreak to state and local health departments.

Since January 2018, CTDPH syndromic surveillance system has collected data on ED visits from all 38 EDs in Connecticut by using the EpiCenter system (Health Monitoring Systems, Inc.). Using Health Level Seven messaging,* EDs transfer visit data (e.g., patient sex, age, ZIP Code of residence, chief complaint, and triage notes) to EpiCenter upon patient registration and discharge in near real-time (i.e., <5 minutes).

Within 20 minutes of receiving the first media report, CTDPH developed an ad hoc syndrome definition to identify ED visits for suspected SC overdoses by querying EpiCenter. The syndrome definition was derived from keywords in the chief complaint, selected in an iterative process from terms in media reports and ED visit record reviews. Initial keywords included terms for SCs (e.g., “K2,” “spice,” or “weed”) and later refined to include terms for location (e.g., “green,” “bench,” or “park”). By midday on August 15, a total of 25 suspected outbreak-related ED visits had been identified; by 5:00 p.m. on August 16, the number had increased to 55, all in New Haven County. CTDPH leadership and the local health department were updated with these data via periodic e-mails. The outbreak response ended on August 17. The U.S. Department of Justice Drug Enforcement Administration determined that SCs implicated in this outbreak contained AMB-FUBINACA, an ultrapotent SC with strong depressant effects (*3*,*4*).

On August 20, CTDPH further refined the syndromic case definition to include keywords in either chief complaint or triage notes to retrospectively identify outbreak-related ED visits during August 15–16 that were missed by near real-time chief complaint analysis. For this retrospective analysis, an outbreak-related ED visit was defined as an ED visit in New Haven County during August 15–16 with SC- or location-related keywords in the chief complaint or triage notes. Among 2,086 ED visits in New Haven County during August 15–16, a total of 72 met the updated outbreak-related SC overdose syndrome definition. Those 72 ED visits comprised 53 unique patients, 12 of whom returned to the ED up to five times for SC overdose visits, indicating possible reexposure to SC containing AMB-FUBINACA. Median patient age was 43 years (interquartile range = 35–51 years), and 41 (77%) patients were male. Among 63 ED visits with discharge disposition data, patients were discharged after 57 ED visits (90%), and six (10%) left without being seen; none died.

Near real-time syndromic surveillance data provided timely situational awareness to public health departments about the approximate magnitude of the outbreak; a follow-up analysis allowed the extent of the SC outbreak to be characterized and confirmed that the outbreak had ended. After this outbreak, CTDPH created additional substance- and location-specific overdose syndrome definitions to help detect future drug overdose–related events and built an exploratory data analysis dashboard to facilitate near real-time data analysis. This outbreak also led to development of CTDPH standard operating guidelines for information sharing and resource allocation with response partners during overdose-related events. CTDPH shared best practices and the syndrome definition from this investigation with the National Syndromic Surveillance Program Community of Practice.^†^ Syndromic surveillance has the potential to be an important tool to provide public health officials with situational awareness of substance use–related morbidity.

## Footnotes

* Health Level Seven is a nationally recognized standard for electronic data exchange between systems housing health care data, which enables two-way exchange of information using a standardized vocabulary and syntax. https://www.cdc.gov/nssp/documents/guides/syndrsurvmessagguide2_messagingguide_phn.pdf.

† The National Syndromic Surveillance Program Community of Practice is a collaboration among CDC, federal partners, state and local health departments, academic institutions, and private sector partners. https://www.cdc.gov/nssp/overview.html.

## References

[1] Riederer AM, Campleman SL, Carlson RG, ; Toxicology Investigators Consortium. Acute poisonings from synthetic cannabinoids—50 U.S. Toxicology Investigators Consortium registry sites, 2010–2015. MMWR Morb Mortal Wkly Rep 2016;65:692–5. 10.15585/mmwr.mm6527a2 27413997 PMC4972329

[2] Vivolo-Kantor AM, Seth P, Gladden RM, Vital signs: trends in emergency department visits for suspected opioid overdoses—United States, July 2016–September 2017. MMWR Morb Mortal Wkly Rep 2018;67:279–85. 10.15585/mmwr.mm6709e1 29518069 PMC5844282

[3] Adams AJ, Banister SD, Irizarry L, Trecki J, Schwartz M, Gerona R. “Zombie” outbreak caused by the synthetic cannabinoid AMB-FUBINACA in New York. N Engl J Med 2017;376:235–42. 10.1056/NEJMoa1610300 27973993

[4] Drug and Chemical Evaluation Section. FUB-AMB (AMB-FUBINACA; MMB-FUBINACA). Springfield, VA: US Department of Justice, Drug Enforcement Administration; 2019. https://www.deadiversion.usdoj.gov/drug_chem_info/FUB-AMB.pdf

